# Effects of Lysozyme and Silver Anode Treatment on *Pseudomonas aeruginosa* Growth and Biofilm Formation in Raw Milk

**DOI:** 10.1111/1750-3841.71179

**Published:** 2026-06-09

**Authors:** Sergul Cibik, Ayhan Duran

**Affiliations:** ^1^ Department of Food Engineering, Faculty of Engineering Aksaray University Aksaray Turkey

**Keywords:** biofilm, lysozyme, *Pseudomonas aeruginosa*, raw milk, silver anode

## Abstract

*Pseudomonas aeruginosa* is an important spoilage‑associated and opportunistic pathogen in raw milk, producing heat‑stable enzymes and robust biofilms that resist conventional sanitation. The limitations of existing decontamination methods necessitate novel, nonthermal, and ecofriendly strategies. This study evaluated the antibacterial and anti‑biofilm effects of lysozyme combined with the silver anode technique (SAT) against *P. aeruginosa* ATCC 27853 in raw milk. The minimum inhibitory concentration (MIC) of lysozyme was estimated by disk diffusion (8 g/100 mL). Raw milk samples were assigned to eight experimental groups: untreated controls, lysozyme alone, SAT alone, and their combination. Bacterial counts (log CFU/mL) and biofilm formation (crystal violet assay) were monitored over 72 h of refrigerated storage, and silver ion migration was quantified by ICP‑MS. Lysozyme alone was predominantly bacteriostatic, while SAT alone produced a bactericidal reduction (up to 1.5–2 log). The combination reduced planktonic counts by up to 2.5 log and biofilm by up to 78%. These reductions were greater than either treatment alone, although the effect was additive, not strictly synergistic. Silver migration (3.88 µg/L) gave a daily intake of 0.055 µg/kg for an adult consuming 1 L, well below EPA (5 µg/kg/day) and EFSA (50 µg/kg food) limits. The combination offers an effective, non‑thermal, eco‑friendly dairy pretreatment strategy. It can extend shelf life, reduce biofilm contamination, and minimize chemical disinfectants, aligning with green technology.

## 1 Introduction

Milk is a highly nutritious food rich in proteins, fats, vitamins, and minerals, but its composition also supports the growth of a wide range of microorganisms, making it prone to microbial spoilage (Başar and Heperkan 2021; Wang et al. 2025). Psychrotrophic bacteria, which can grow under refrigeration, pose a serious threat to the shelf life of raw milk and the quality of processed dairy products.

Among these psychrotrophic bacteria, *Pseudomonas aeruginosa* is a particularly problematic species for the dairy industry. This Gram‐negative bacterium, which is widespread in nature, can easily contaminate milk through unhygienic milking conditions (e.g., dirty udders, insufficiently cleaned milking equipment, poor hand hygiene of milkers) and equipment, and can multiply rapidly during cold storage, often becoming a significant part of the psychrotrophic flora (Şen and Halkman 2006). One of the most significant risks posed by *P. aeruginosa* is its production of heat‐resistant protease and lipase enzymes that cannot be inactivated by conventional heat treatment. These enzymes have been shown to cause irreversible quality defects, such as rancidity and gel formation (“sweet curdling”), even in pasteurized and UHT‐treated milk during storage (SahityaRani 2025).

Nevertheless, the primary challenge posed by *P. aeruginosa* arises from the biofilm structures it forms by adhering to surfaces. A biofilm may be defined as a structured community of bacteria that is held together within an exopolysaccharide (EPS) matrix produced by the bacteria themselves. This structure functions as a formidable barrier, providing protection to the bacteria against disinfectants, antimicrobial agents, and environmental stresses (Donlan and Costerton 2002). The intricate regulation of biofilm formation is orchestrated by an intercellular communication system known as “quorum sensing” (QS). The system in question is initiated upon the attainment of a specific population density threshold, thereby inducing collective behaviors such as biofilm formation and secretion of virulence factors (Striednig and Hilbi 2022). Consequently, the formation of *P. aeruginosa* biofilms in hard‐to‐clean areas, such as pipe surfaces, gaskets, and tanks in dairy processing lines, establishes a persistent source of contamination, thereby jeopardizing product quality and safety (Kütük and Temiz 2022).

The ineffectiveness of conventional cleaning and disinfection protocols against resistant structures has necessitated the development of innovative and alternative control strategies. In this context, natural antimicrobials and physicochemical interventions offer significant potential. Although lysozyme is primarily recognized as an enzyme that degrades the cell wall of Gram‐positive bacteria, studies have demonstrated its efficacy against Gram‐negative bacteria (Masschalck and Michiels 2003). It has been reported that lysozyme, due to its positively charged structure, can interact with the bacterial cell membrane. Based on these observations, some researchers have suggested that lysozyme might play a role in inhibiting *P. aeruginosa* biofilm formation (Hukić et al. 2018; Eladawy et al. 2020).

Another promising approach is to utilize the antimicrobial property of silver. Silver ions (Ag^+^) exhibit an oligodynamic effect, resulting in a broad antimicrobial spectrum even at very low concentrations. These ions have been shown to inhibit the respiratory enzymes of the bacterial cell, disrupt membrane permeability, and interact with nucleic acids, ultimately resulting in cell death (Secinti et al. 2016; Khalandi et al. 2017). The silver anode technique (SAT) facilitates the regulated ionization of silver by means of an electric current, thereby enabling the attainment of this potent effect within a specific environment and over an extended duration (Aydin et al. 1997; Cibik and Duran 2022). As demonstrated in the extant literature, SAT has been shown to be effective in combating both planktonic cells and biofilm structures of *P. aeruginosa* (Dusane et al. 2019; Cibik and Duran 2022). Although the SAT itself is not classified as GRAS, silver is regulated as a food contact substance by the US FDA and by the European EFSA with a specific migration limit (EFSA CEF Panel 2011). From an environmental perspective, the technique offers potential green benefits (reduced chemical use, low energy consumption), but its overall sustainability depends on proper silver recovery and waste management (Mijnendonckx et al. 2013).

Whilst the extant literature has focused on the individual antimicrobial effects of lysozyme and silver, the potential of combining these two agents with different mechanisms of action has not been sufficiently investigated, especially in a complex food matrix such as raw milk. We hypothesize that the damage lysozyme may cause to the cell surface and outer membrane could facilitate the entry of silver ions into the cell and enhance their toxic effect, thereby offering a strategy for controlling biofilm structures resistant to conventional methods.

The primary hypothesis of this study is that the combination of lysozyme and SAT will inhibit the biofilm formation and viability of *P. aeruginosa* in raw milk to a greater extent than either method alone. To test this, the study had three specific objectives: (1) to determine the minimum inhibitory concentration (MIC) of lysozyme against *P. aeruginosa*; (2) to establish the optimal current and application time for SAT; and (3) to comparatively evaluate the anti‐biofilm and antibacterial effects of the two methods, individually and in combination, on natural and inoculated *P. aeruginosa* flora during refrigerated storage (initial, 24, 48, 72 h).

## 2 Materials and Methods

### 2.1 Bacterial Strain and Culture Conditions

The study utilized the standard reference strain *P. aeruginosa* ATCC 27853. The bacterium was activated from a 20% glycerol stock by inoculating into 10 mL of tryptic soy broth (TSB) medium (105459, SIGMA) and incubating at 37°C for 24 h. Confirmation of the active culture was performed by oxidase‐positive reaction and a growth test on Cetrimide Agar (CA) (105284, MERCK) at 42°C, which is specific to *P. aeruginosa* (Al‐Momani et al. 2024).

### 2.2 Raw Milk Samples and Chemical Analyses

Raw cow's milk was obtained from the Cattle Breeders' Association under aseptic conditions (i.e., collected directly into sterile containers using hygienic milking practices). Baseline microbiological analysis confirmed the presence of natural *P. aeruginosa* flora at an initial count of approximately 2.08 log CFU/mL (Table 2, Group C). To ensure the representativeness and reproducibility of the results, milk was collected in three independent batches from different collection days. Upon arrival at the laboratory, the milk samples from each batch were immediately processed and analyzed. Basic compositional analyses of the milk (fat, moisture, protein, lactose, and pH) were performed in triplicate using AOAC methods (AOAC 2005). Protein analysis was performed in accordance with the Kjeldahl method (AOAC 2005; Method 991.20), employing a correction factor of 6.38. The carbohydrate (lactose) content was determined according to the Lane–Eynon method (Hansen and Ferrao 2018), and pH was measured using a pH meter (pH 211, Hanna Instruments) (El‐Saadony et al. 2021).

### 2.3 Determination of MIC for Lysozyme

The antibacterial activity of lysozyme (MAYSA, Türkiye; ≥ 35,000 U/mg, from egg white) against *P. aeruginosa* ATCC 27853 was assessed using the Kirby–Bauer disk diffusion method (Bauer et al. 1966; Loho et al. 2018), following the guidelines of the Clinical and Laboratory Standards Institute (CLSI M02). Lysozyme solutions were prepared in sterile 0.9% NaCl at concentrations ranging from 0.125 to 16 g/100 mL. A 10 µL aliquot of each lysozyme solution was impregnated onto sterile blank paper disks (6 mm diameter, BİOANALYSE, Türkiye). Mueller–Hinton Agar (MHA) plates were inoculated with a bacterial suspension adjusted to the 0.5 McFarland standard (approximately 1.5 × 10^8^ CFU/mL). The impregnated disks were placed on the MHA surface and incubated at 37°C for 24 h. Following incubation, the diameters of the inhibition zones were measured using a digital caliper. The lowest concentration that produced a measurable inhibition zone (≥ 7 mm) was recorded as the MIC, which was determined to be 8 g/100 mL.

### 2.4 Setup of the SAT

**FIGURE 1:**
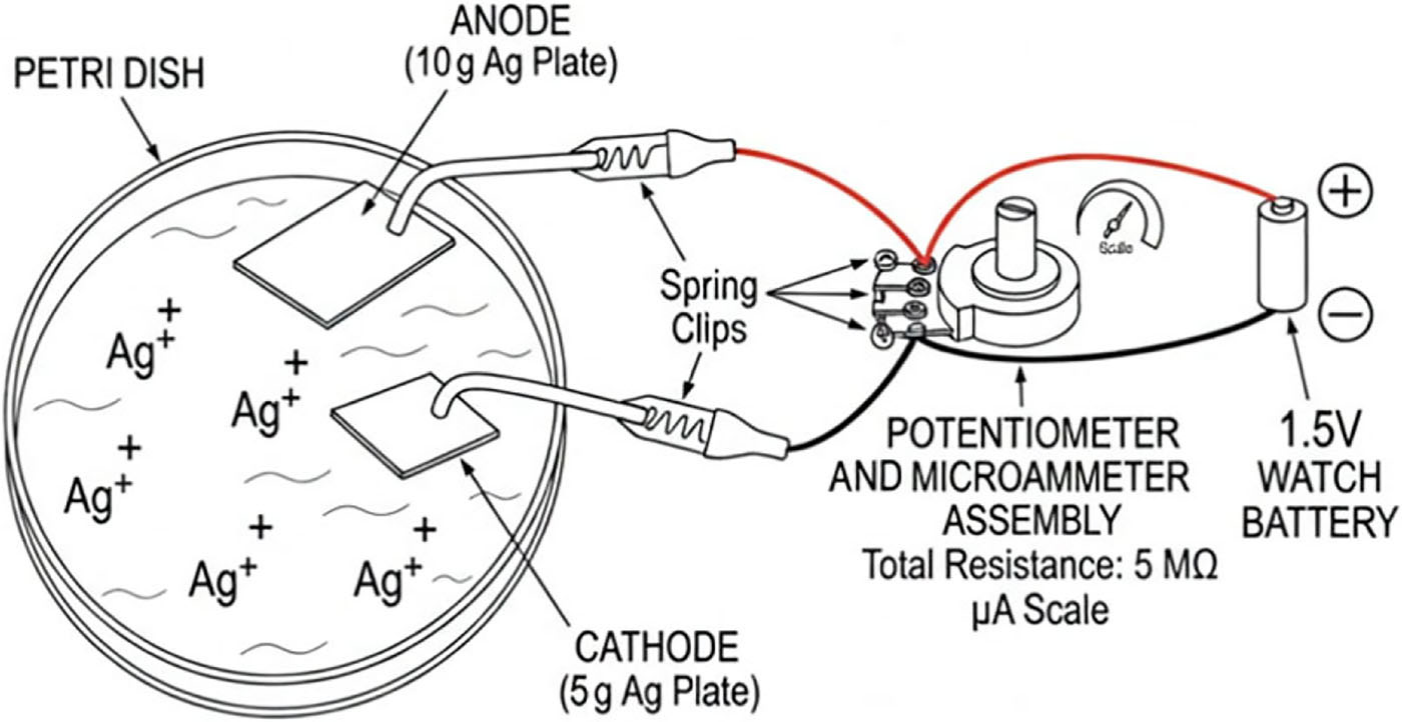
Setup and application of the silver anode technique (SAT).

The SAT setup consisted of a 1.5 V clock battery as the power source, a 5 MΩ potentiometer to regulate the current, and two silver plates with 99.9% purity. The anode weighed 10 g and measured 25 × 15 × 3 mm^3^, while the cathode weighed 5 g and measured 23 × 14 × 1.5 mm^3^. Both electrodes were equipped with 8 mm‐high stems to prevent direct contact with the Petri dish bottom, ensuring maximum surface exposure to the milk (Çibik and Duran 2022). They were positioned symmetrically within the 9 cm‐diameter Petri dish, maintaining a constant distance of approximately 5 cm between them throughout all applications. The required resistance for the desired current (20 µA, as optimized for *P. aeruginosa* ATCC 27853) was calculated using Ohm's Law (*I* = *V*/*R*) after measuring the battery voltage with a multimeter. For a measured battery voltage of 1.361 V, the potentiometer was set to 0.068 MΩ to achieve the target current of 20 µA. The system was then connected, and the current was applied to the raw milk samples for 60 s. Figure 1 illustrates the complete experimental arrangement.

### 2.5 Experimental Design and Application

Eight different experimental designs were created to evaluate the individual and combined effects of lysozyme and SAT:

‐ C: 40 mL raw milk (negative control)
‐ C1: 39 mL raw milk + 1 mL bacterial suspension (positive control)
‐ C2: 40 mL raw milk + Lysozyme (MIC value)
‐ C3: 39 mL raw milk + Lysozyme (MIC value) + 1 mL bacterial suspension
‐ C4: 40 mL raw milk + SAT (optimal parameters)
‐ C5: 39 mL raw milk + 1 mL bacterial suspension + SAT (optimal parameters)
‐ C6: 39 mL raw milk + Lysozyme (MIC value) + 1 mL bacterial suspension + SAT (optimal parameters)
‐ C7: 40 mL raw milk + Lysozyme (MIC value) + SAT (optimal parameters)

All applications were carried out in 40 mL sterile glass Petri dishes. For treatment groups that received 1 mL of bacterial suspension (C1, C3, C5, C6), the volume of raw milk was adjusted to 39 mL to maintain a constant final volume of 40 mL.

### 2.6 Evaluation of Antibacterial Activity

Samples were collected from the milk in all experimental designs immediately following application (Initial) and at 24, 48, and 72 h of storage at +4°C. The samples were serially diluted with sterile 0.9% NaCl and plated onto CA medium using the spread plate method. CA is a selective medium that specifically supports the growth of *P. aeruginosa* while inhibiting most other microorganisms. To ensure accurate quantification, all colonies grown on CA were presumptively identified as *P. aeruginosa* based on their typical morphology (flat, spreading, greenish‐pigmented colonies) and confirmed by oxidase‐positive reaction and growth at 42°C (specific for *P. aeruginosa*), as described in Section 2.1 (Al‐Momani et al. 2024). The Petri dishes were incubated at 35 ± 2°C for 48 h, and the resulting colonies were counted and expressed as log_10_ CFU/mL. This incubation regime is that originally described by Mossel and Indacochea (1971) for the selective enumeration of *P. aeruginosa* on CA.

### 2.7 Biofilm Analysis

The measurement of biofilm formation was conducted in accordance with the microtiter plate method, as modified by O'Toole (2011). In summary, 96‐well microtiter plates were inoculated using two different volume ratios to ensure adequate biofilm formation. For the first set of wells, 180 µL of TSB_G_ (TSB supplemented with 1% glucose) was mixed with 20 µL of the sample (final volume 200 µL). For the second set, 100 µL of TSB_G_ was mixed with 100 µL of the sample (final volume 200 µL). Pilot experiments showed that the 100 + 100 µL ratio consistently produced more robust biofilm growth, so absorbance values obtained from this setup were used for subsequent analyses. The plates were then incubated at 37°C for 24 h. This temperature is the established optimum for the human pathogen *P. aeruginosa* ATCC 27853 (ATCC 2025) and is standardly used in microtiter plate biofilm assays for this species (Naves et al. 2008; O'Toole 2011).

**FIGURE 2:**
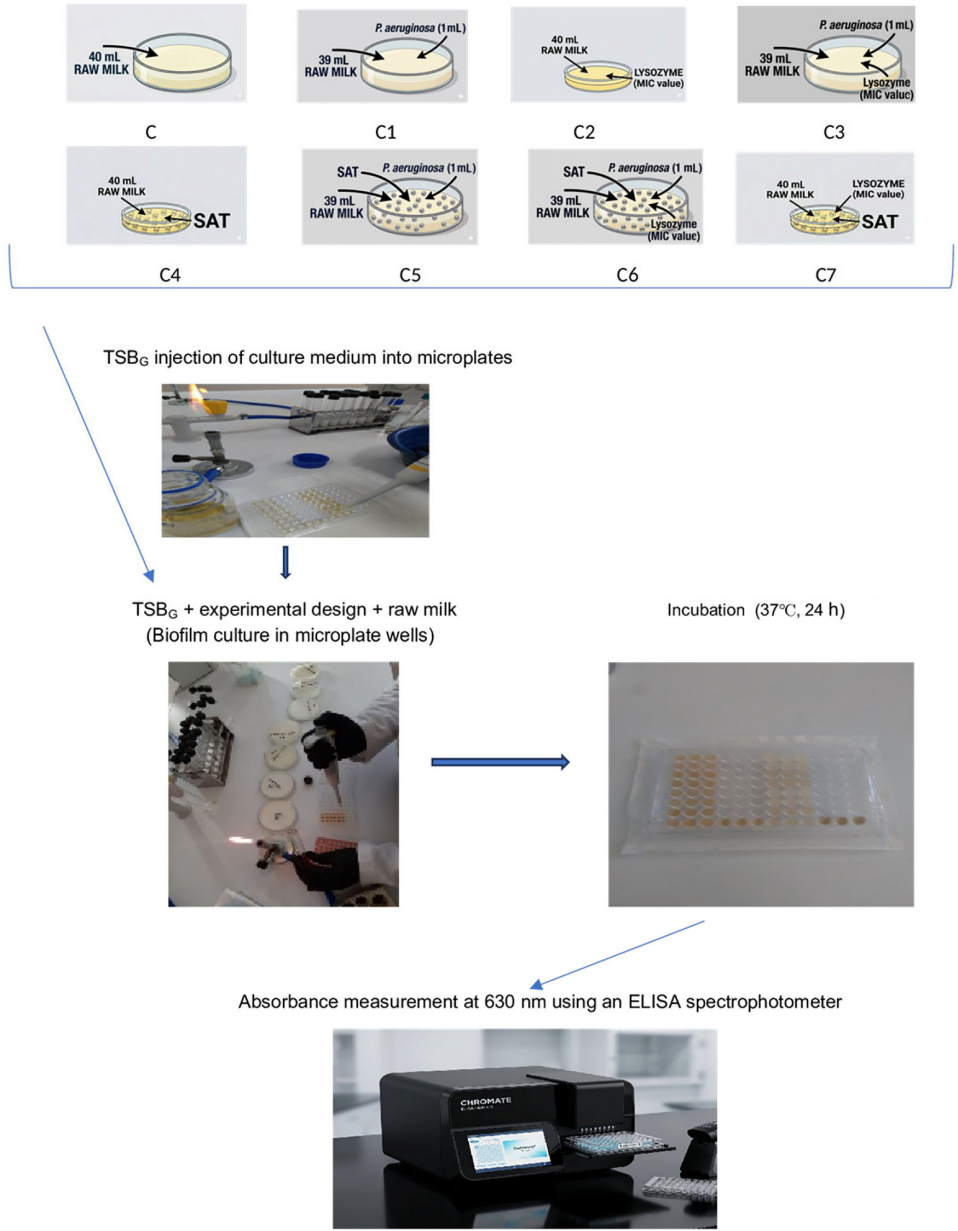
Schematic overview of the experimental biofilm analysis procedure.

The percentage inhibition of biofilm formation for each treatment was calculated relative to its respective control using the following formula (Naves et al. 2008):

$$\%\;\mathrm{Biofilm}\;\mathrm{Inhibition}=100\times \left(OD_{\mathrm{control}}-OD_{\mathrm{treatment}}\right)/OD_{\mathrm{control}}$$

where OD_control_ is the average absorbance of the positive control wells (C1 for inoculated groups; C for natural flora groups) and OD_treatment_ is the average absorbance of the treated wells. Thus, a higher percentage indicates a greater reduction in biofilm formation. The preparation of microplate wells after incubation is shown in Figure 2.

### 2.8 Determination of Silver Ion (Ag^+^) Migration Into Milk

Silver ion migration in milk samples treated with SAT was analyzed using Inductively Coupled Plasma Mass Spectrometry (ICP‐MS, Thermo Scientific X‑Series II, USA). Samples were subjected to microwave digestion using concentrated HNO_3_ and H_2_O_2_ in a closed‐system unit, followed by dilution with ultrapure water, and preparation for analysis (Dokprom et al. 2025).

### 2.9 Statistical Analysis

The study employed a completely randomized design with eight experimental groups (C, C1, C2, C3, C4, C5, C6, C7), each tested in triplicate across four storage time points (initial, 24, 48, 72 h). Colony counts obtained from the antibacterial activity studies were performed in triplicate, and the data were subjected to one‐way analysis of variance (ANOVA) using SPSS 22.0 (SPSS Inc., USA) software. The differences between the means were evaluated using Duncan's multiple range test at a 95% confidence level (*p* < 0.05) (Snedecor and Cochran 1980).

For biofilm formation, the mean absorbance values (OD_avg_) from three independent experiments were also analyzed by one‐way ANOVA followed by Duncan's post hoc test to determine significant differences among treatment groups (Table 4). The percentage inhibition of biofilm formation (Table 5) was then calculated as described in Section 2.7. Silver ion migration data were obtained from triplicate ICP‐MS measurements and are reported as mean ± standard deviation (SD); no further inferential statistical tests were applied, as the objective was to compare the detected silver concentration directly with the established EPA safety limit (EPA 1991).

## 3 Results and Discussion

### 3.1 Chemical Analysis Results

The chemical analysis results of the raw milk used in the study are presented in Table 1. The composition (fat 3.78%, protein 3.50%, lactose 4.46%, moisture 87.32%, pH 6.68) was within the typical ranges for raw cow milk and complied with the local standard for raw milk (TS 1018/T4 2025). These values confirm that the milk matrix used in this study was representative of commercially available raw milk, ensuring the relevance of the experimental findings.

**TABLE 1:** Chemical analysis results of raw milk.

Fat%	3.78 ± 0.01
Protein%	3.50 ± 0.03
Lactose%	4.46 ± 0.03
Moisture%	87.32 ± 0.05
pH	6.68 ± 0.01

*Note*: Values are mean ± SD of three independent milk batches.

### 3.2 Determination of Lysozyme MIC and Silver Anode Parameters

As a consequence of disk diffusion tests conducted against the *P. aeruginosa* ATCC 27853 strain, the MIC value for the lysozyme enzyme was ascertained to be 8 g/100 mL (equivalent to 8% w/v). This relatively high concentration is indicative of the significant challenges faced by lysozyme in penetrating the outer membrane of *P. aeruginosa*, a Gram‐negative bacterium. Gram‐negative bacteria, due to their lipopolysaccharide (LPS) outer membrane structure, physically restrict the access of lysozyme to its target, the peptidoglycan layer (Vaara 2020). However, it has been documented in the extant literature that at elevated concentrations, due to its cationic nature, lysozyme can cause disruption in the outer membrane, exhibiting bacteriostatic and, under certain conditions, bactericidal effects (Pellegrini et al. 1992; Deckers et al. 2008). It is hypothesized that this mechanism may also prove effective in the present study.

In our previous Taguchi optimization study (Cibik and Duran 2023), the most effective antibacterial effect against *P. aeruginosa* ATCC 27853 in raw milk (a reduction of 1.31 log CFU/mL) was achieved with a current of 20 µA and an application time of 60 s. In that study, four distinct current levels (5, 10, 15, 20 µA) and four varying application times (15, 30, 45, 60 s) were examined in raw milk inoculated with *P. aeruginosa* ATCC 27853, and an L_16_ orthogonal array was constructed. Treated samples were plated on CA medium, incubated at 35 ± 2°C for 48 h, and the developed colonies were enumerated and converted to log10 CFU/mL. The optimum current and time were determined by calculating signal‐to‐noise (S/N) ratios based on the “smaller‐the‐better” approach. This finding demonstrated that SAT is effective even at low current levels. Aydin et al. (1997) similarly stated that currents above 20 µA do not significantly increase the antibacterial effect and may even lead to undesirable side effects with uncontrolled applications. These optimized parameters were therefore adopted for all SAT treatments in the present study. They also serve as an important guide for energy efficiency and application safety in industrial settings.

### 3.3 Changes in Antibacterial Activity During Storage

The antibacterial effects of lysozyme and SAT, alone and in combination, on raw milk samples were monitored throughout 72 h of cold storage (Tables 2 and 3).

**TABLE 2:** Antibacterial effect of lysozyme and SAT on *Pseudomonas aeruginosa* in raw milk.

	Initial	24th hour	48th hour	72nd hour
*P. aeruginosa* (log CFU/mL)				
C	2.08 ± 0.17^d1^	2.33 ± 0.05^c1^	2.48 ± 0.40^b1^	2.70 ± 0.55^a1^
C2	2.07 ± 0.01^c1^	2.21 ± 0.02^b2^	2.36 ± 0.05^a2^	2.25 ± 0.01^b2^
C4	1.26 ± 0.03^a2^	1.18 ± 0.02^b3^	1.13 ± 0.04^c3^	1.11 ± 0.02^c3^
C7	1.26 ± 0.01^a2^	1.06 ± 0.01^b4^	0.86 ± 0.03^c4^	0.54 ± 0.09^d4^

*Note*: Different superscript letters within a row indicate a significant difference (*p* < 0.05). Different superscript numbers within a column indicate a significant difference (*p* < 0.05). C: raw milk (negative control); C2: raw milk + lysozyme; C4: raw milk + SAT; C7: raw milk + lysozyme + SAT.

**TABLE 3:** Antibacterial effect of lysozyme and SAT on *Pseudomonas aeruginosa* ATCC 27853 inoculated into raw milk.

	Initial	24th hour	48th hour	72nd hour
*P. aeruginosa* ATCC 27853 (log CFU/mL)				
C1	5.57 ± 0.03^d1^	5.93 ± 0.02^c1^	6.15 ± 0.04^b1^	6.45 ± 0.03^a1^
C3	5.56 ± 0.04^c1^	5.62 ± 0.04b^c2^	5.68 ± 0.04^ab2^	5.70 ± 0.03^a2^
C5	4.53 ± 0.05^a2^	4.44 ± 0.02^b3^	4.40 ± 0.02^b3^	4.39 ± 0.06^b3^
C6	4.52 ± 0.03^a2^	4.26 ± 0.01^b4^	4.09 ± 0.02^c4^	3.92 ± 0.02^d4^

*Note*: Different superscript letters within a row indicate a significant difference (*p* < 0.05). Different superscript numbers within a column indicate a significant difference (*p* < 0.05). C1: raw milk + *P. aeruginosa* ATCC 27853 (positive control); C3: C1 + lysozyme; C5: C1 + SAT; C6: C1 + lysozyme + SAT.

In the designs where lysozyme was applied in isolation (C2 and C3), no significant increase in the bacterial population was observed during storage in comparison to the control group (C1). However, no sharp decrease in log CFU/mL values was observed either. This finding suggests that lysozyme exerts primarily a bacteriostatic effect on *P. aeruginosa*. This finding can be interpreted as lysozyme suppressing bacterial growth by inhibiting cell division or slowing down metabolism, but being insufficient to completely kill existing cells (Ibrahim et al. 2001).

Conversely, in the designs where SAT was applied alone (C4 and C5), an approximate 1 log reduction was detected immediately after the application (Initial), and this effect reached a 1.5–2 log reduction by the 72nd hour of storage. This dramatic decrease is indicative of the profound bactericidal effect of SAT. The multi‐targeted mechanism of action of silver ions (Ag^+^) is considered the primary reason for this strong and persistent bactericidal activity. This mechanism involves the inhibition of respiratory enzymes, the disruption of membrane integrity, and the prevention of DNA replication in the bacterial cell (Percival et al. 2005; Secinti et al. 2016). Furthermore, the “residual effect” of silver ions present in the milk even after the electric current has been terminated (Spadaro 1985) may have been a contributing factor to the sustained efficacy during storage.

The largest reduction in bacterial counts was observed in the designs where lysozyme and SAT were applied in combination (C6 and C7). The combination of these factors resulted in a reduction of over 1 log at the time of application, leading to a total bacterial reduction of 2–2.5 log CFU/mL by the 72nd hour of storage. The combination produced a greater reduction than either lysozyme or SAT alone. However, when comparing the observed reduction (2.53 log CFU/mL at 72 h in inoculated milk) with the sum of the individual reductions (0.75 log for lysozyme + 2.06 log for SAT = 2.81 log), the combined effect did not exceed the additive sum. Therefore, the effect is best described as additive to slightly enhanced, rather than strictly synergistic.

The following section will explore a potential mechanism of synergism. The transient damage or disruption caused by lysozyme to the outer membrane of *P. aeruginosa* due to its cationic nature (Deckers et al. 2008) may have facilitated the entry of silver ions into the cell and their access to target sites. In summary, while lysozyme weakens the bacterium's “defense shield,” and SAT exploits this vulnerability to exert its bactericidal effect. The combination of these two distinct mechanisms (membrane destabilization by lysozyme and multiple intracellular damages by silver ions) produced a superior bactericidal effect compared to either mechanism alone, even against bacteria within biofilm structures that are resistant to conventional methods.

### 3.4 Effects on Biofilm Inhibition

**FIGURE 3:**
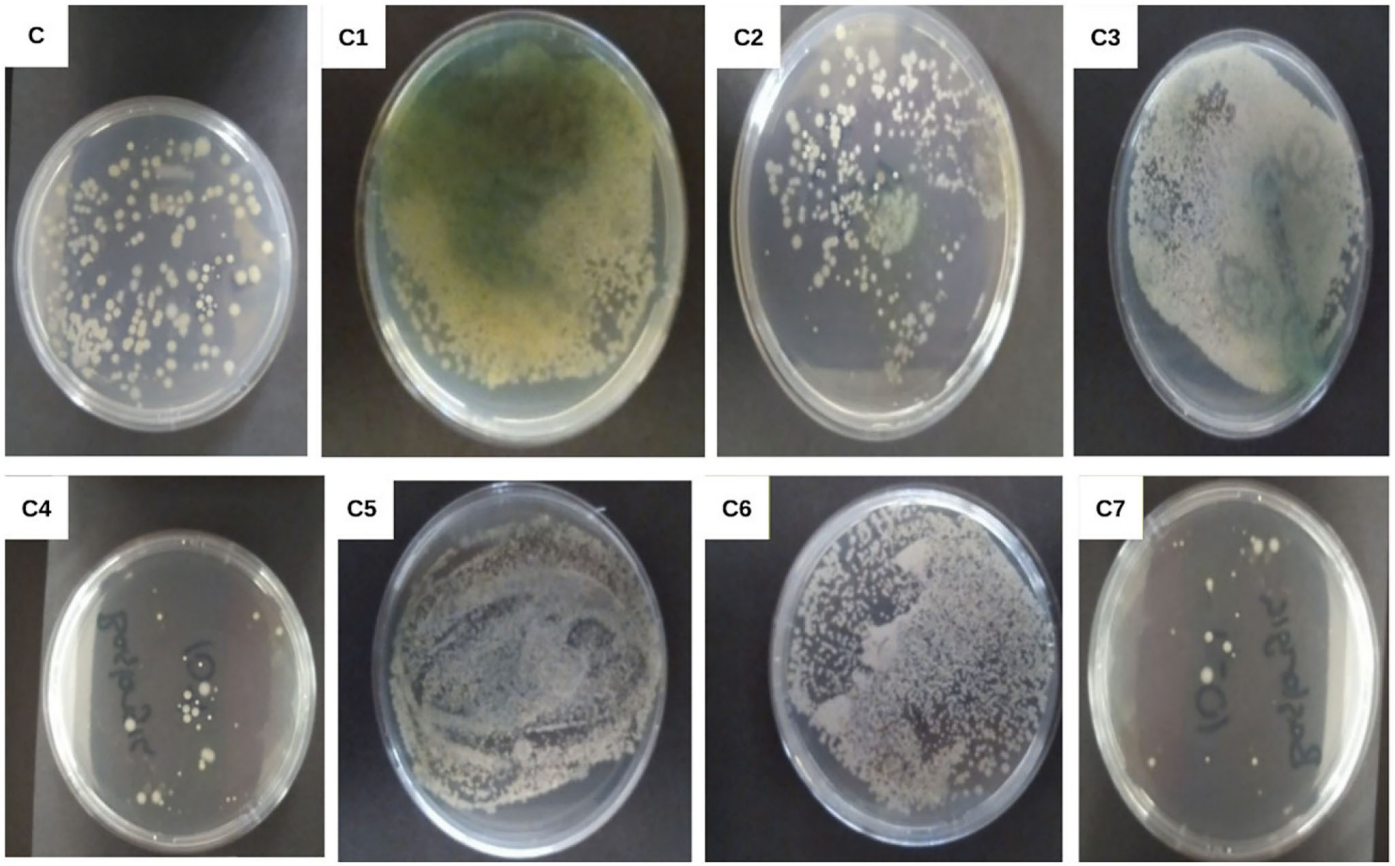
Colony formation results of the 10^−1^ dilution at the initial time. Each Petri dish is labeled with its corresponding experimental code (C, C1, C2, C3, C4, C5, C6, and C7).

Representative colony formation results at the initial time (10^−1^ dilution) for all experimental groups are presented in Figure 3. Biofilm analysis results supported the antibacterial activity findings and showed that the combination was even more effective on the biofilm layer (Tables 4 and 5).

**TABLE 4:** Absorbance (OD_630_) values and biofilm formation characteristics of experimental groups.

Application design	Absorbance (OD) values				Biofilm formation characteristic
	OD_1_	OD_2_	OD_3_	OD_avg_	
C	0.524	0.523	0.529	0.525	++ (moderate)
C1	1.051	1.036	1.040	1.042	+++ (strong)
C2	0.274	0.276	0.281	0.277	+ (weak)
C3	0.624	0.623	0.629	0.625	++ (moderate)
C4	0.197	0.198	0.198	0.198	− (none)
C5	0.439	0.441	0.440	0.440	+ (weak)
C6	0.232	0.231	0.233	0.232	− (none)
C7	0.133	0.134	0.133	0.133	− (none)
Positive control (*Pseudomonas aeruginosa* 27853)	1.467	1.468	1.472	1.469	+++ (strong)
Negative control (sterile TSB_G_)	0.232	0.234	0.231	0.232	− (none)

*Note*: OD cut‑off value = 0.234 (mean of negative control + 2 × SD). Biofilm interpretation: −(none, OD ≤ 0.234), + (weak, 0.234 < OD ≤ 0.470), ++ (moderate, 0.470 < OD ≤ 0.940), +++ (strong, OD > 0.940). C: raw milk; C1: C + *P. aeruginosa*; C2: C + lysozyme; C3: C1 + lysozyme; C4: C + SAT; C5: C1 + SAT; C6: C1 + lysozyme + SAT; C7: C + lysozyme + SAT.

**TABLE 5:** Biofilm inhibition (%) by different treatments compared to respective controls.

Control group (OD_initial_)	Treatment group (OD_final_)	Biofilm inhibition (%)^a^	Interpretation
C (natural flora)	C2 (lysozyme)	47 ± 3^b^	Moderate reduction
C (natural flora)	C4 (SAT)	62 ± 2^a^	High reduction
C (natural flora)	C7 (lysozyme + SAT)	75 ± 2^a^	Very high reduction
C1 (inoculated)	C3 (lysozyme)	40 ± 2^b^	Moderate reduction
C1 (inoculated)	C5 (SAT)	58 ± 3^a^	High reduction
C1 (inoculated)	C6 (lysozyme + SAT)	78 ± 2^a^	Very high reduction

*Note*: C: raw milk; C1: C + *P. aeruginosa*; C2: C + lysozyme; C3: C1 + lysozyme; C4: C + SAT; C5: C1 + SAT; C6: C1 + lysozyme + SAT; C7: C + lysozyme + SAT. ^a‐b^ Different superscript letters within a column indicate significant difference (*p* < 0.05).

^a^ %Biofilm Inhibition was calculated as 100 × (OD_control_ − OD_treatment_) / OD_control_. Positive values indicate a reduction in biofilm formation compared to the respective control.

Lysozyme alone (C2 for natural flora, C3 for inoculated) inhibited biofilm formation by 47% (natural) and 40% (inoculated). SAT alone (C4 for natural flora, C5 for inoculated) achieved higher inhibition rates of 62% (natural) and 58% (inoculated). The enhanced anti‐biofilm efficacy of SAT can be attributed to the capacity of silver ions to permeate the EPS matrix, thereby eradicating bacterial cells within it (Kalishwaralal et al. 2010). The combination of lysozyme and SAT (C7 for natural flora, C6 for inoculated) was the most effective, with inhibition rates of 75% (natural) and 78% (inoculated).

This finding demonstrates the efficacy of the synergistic mechanism not only on planktonic (free‐swimming) cells but also on cells encased within the protective biofilm matrix. The interference of lysozyme with the extracellular polymers or cell–cell interactions that stabilize the biofilm structure may have enabled SAT to penetrate the biofilm more deeply. This finding offers considerable potential for the utilization of this combination in industrial cleaning and disinfection protocols, particularly for the treatment of surfaces covered with biofilms.

### 3.5 Safety Assessment: Silver Migration Into Milk

ICP‐MS analysis showed that the amount of silver ions (Ag^+^) migrating into raw milk after a single SAT treatment (20 µA, 60 s) was 3.882 ± 0.01 µg/L.

To place this value in a risk context: if an adult (70 kg body weight) consumes 1 L of such milk, the ingested silver would be 3.88 µg, corresponding to 0.055 µg/kg body weight/day. This is well below the oral reference dose established by the US Environmental Protection Agency (EPA 1991), which is 5 µg/kg/day. Furthermore, this level is approximately 13 times lower than the group‐specific migration limit of 50 µg silver/kg food (0.05 mg/kg) established for silver in food contact materials by the European Food Safety Authority (EFSA CEF Panel 2011), a limit also reaffirmed in EFSA's later assessment of silver nanoparticles (EFSA CEP Panel et al. 2021). Even at a higher consumption level (e.g., 250 mL for a child), the exposure remains minimal.

It is important to note, however, that this measurement reflects a single application under static laboratory conditions. The data do not account for potential accumulation of silver from repeated treatments (e.g., in a continuous industrial process) or for long‐term consumption of milk treated this way. Furthermore, comparison with silver migration studies from food packaging (Echegoyen and Nerín 2013; von Goetz et al. 2013) is not fully parallel, because packaging involves passive, continuous release over a product's shelf life, whereas our SAT is a one‐time, short, electrochemically driven intervention.

In conclusion, the measured Ag^+^ level of 3.882 µg/L corresponds to a daily intake of 0.055 µg/kg body weight for an adult consuming 1 L of treated milk, which is two orders of magnitude below the EPA oral reference dose (5 µg/kg/day). This demonstrates that the optimized SAT parameters produce a very low silver release. Nevertheless, because this measurement was obtained under single‐application, laboratory‐scale conditions, further studies are required to assess potential accumulation under repeated or industrial‐scale applications and to establish a comprehensive safety framework. Under the tested conditions, the detected Ag^+^ level is remarkably low and well within current safety limits.

### 3.6 General Evaluation and Industrial Implications

The findings demonstrate that combining lysozyme with the SAT is markedly more effective against both planktonic and biofilm forms of *P. aeruginosa* in raw milk than either treatment alone. This enhanced efficacy stems from the complementary mechanisms of action: lysozyme destabilizes the Gram‐negative outer membrane, facilitating deeper penetration of electrochemically generated silver ions, which then exert multi‐target bactericidal effects.

Based on these results—a reduction of up to 2.5 log CFU/mL in planktonic cells and up to 78% inhibition of biofilm formation—the lysozyme + SAT combination can be recommended as an environmentally friendly, non‐thermal pretreatment strategy for dairy processing plants. Specifically, this approach can extend the shelf life of raw milk by controlling psychrotrophic spoilage organisms, reduce the risk of persistent biofilm‐related contamination in pipelines and storage tanks, limit the formation of heat‐resistant proteolytic and lipolytic enzymes that degrade final product quality, and minimize the use of conventional chemical disinfectants along with their associated residue risks.

Overall, this combined strategy offers a novel contribution to food safety and aligns with sustainable production goals by reducing chemical inputs while maintaining or improving microbiological control.

## 4 Conclusions

This laboratory‐scale study successfully demonstrated that the combination of lysozyme and the SAT produced a clear and substantial antibacterial and anti‐biofilm effect against *P. aeruginosa* in raw milk. The combined treatment reduced planktonic bacterial counts by up to 2.5 log CFU/mL and inhibited biofilm formation by up to 78%, both of which were significantly greater than the reductions achieved by either treatment alone. These results highlight the potential of this dual approach as a non‐thermal, environmentally friendly intervention strategy for dairy applications. The observed enhancement can be explained by complementary mechanisms: the cationic, membrane‐destabilizing action of lysozyme likely facilitates the entry of electrochemically generated silver ions into bacterial cells, thereby increasing their lethal effect. This combination thus transforms the effect from a predominantly bacteriostatic one (lysozyme alone) to a clearly bactericidal one.

The findings carry meaningful implications for the dairy industry. The lysozyme + SAT combination offers a pragmatic and eco‐friendly alternative to conventional chemical sanitizers, with the potential to extend raw milk shelf life, reduce biofilm‐related contamination in processing equipment, limit the formation of heat‐resistant spoilage enzymes, and minimize chemical disinfectant residues. Furthermore, the use of a low‐current SAT ensures energy efficiency, while lysozyme is a natural enzyme already present in milk and egg white, making this strategy consistent with green technology principles.

Nevertheless, certain limitations should be acknowledged to properly contextualize the conclusions. This study used a single reference strain (*P. aeruginosa* ATCC 27853) under static, Petri‐dish conditions, not on industrial surfaces or under continuous flow. Formal synergy testing (e.g., checkerboard or isobologram methods) was not performed, so the interaction is best described as additive to slightly enhanced rather than strictly synergistic. Silver migration was measured only for a single application; repeated or continuous use scenarios were not evaluated. Pilot‐scale and industrial validation remain to be conducted.

Despite these limitations, the positive results obtained under controlled conditions provide a strong rationale for further development. Future research should include testing against field isolates and multi‐species biofilms, evaluating efficacy on stainless steel surfaces under flow, performing formal synergy analyses, assessing silver accumulation under repeated applications, and conducting pilot‐scale trials. In conclusion, the combination of lysozyme and SAT represents a promising, innovative, and sustainable strategy for controlling *P. aeruginosa* in raw milk, meriting continued investigation toward potential industrial application.

## Author Contributions

**Sergul Cibik**: software, resources, data curation, writing – review and editing, visualization, writing – original draft. **Ayhan Duran**: software, formal analysis, project administration, supervision, resources, writing – review and editing, visualization, validation, methodology, conceptualization.

## Funding

The authors have nothing to report.

## Conflicts of Interest

The authors declare no conflicts of interest.

## Data Availability

The data supporting the findings of this study are available from the corresponding author upon reasonable request.
